# Development of blueprint materials that strengthen and embed the infection control link nurse role in hospitals – an action research study

**DOI:** 10.1186/s43058-026-00942-x

**Published:** 2026-04-18

**Authors:** Mireille Dekker, Christiaan Vis, Femke van Nassau, Per Nilsen, Rosa van Mansfeld, Irene P. Jongerden

**Affiliations:** 1 Department of Medical Microbiology and Infection Prevention, Amsterdam UMC, Vrije Universiteit Amsterdam, Amsterdam Public Health Research Institute, Boelelaan 1117, Amsterdam, The Netherlands; 2 Department of Public and Occupational Health, Amsterdam UMC, Vrije Universiteit Amsterdam, Amsterdam Public Health Research Institute, Amsterdam, The Netherlands; 3 Department of Health, Medicine and Caring Sciences, Linköping University, Linköping, 58183 Sweden; 4 Department of Medical Microbiology and Infection Prevention, Amsterdam Institute for Immunology and Infectious Diseases, Amsterdam UMC, Vrije Universiteit Amsterdam, Boelelaan 1117, Amsterdam, The Netherlands

**Keywords:** Complex Intervention, Implementation strategy, Infection Prevention and Control, Nursing, Stakeholders, Co-creation

## Abstract

**Background:**

In hospitals, infection control link nurses (ICLNs) serve as a bridge between their peers and the infection prevention team, driving infection prevention measures through motivation, practical guidance, and knowledge sharing. The success of the ICLN role varies depending on how well it is supported and integrated into hospital structures. In this study, we co-created materials for infection control practitioners (ICPs), incorporating activities to support ICLNs with strategies to strengthen and embed the ICLN role in hospitals. We aimed to develop materials to support and implement the role, and evaluated how cocreation contributed to its normalization in participating hospitals.

**Methods:**

We used an action research approach with co-creation as a general guiding principle. Stakeholders and end-users from ten participating hospitals collaboratively developed blueprint materials, including a role description, training resources and strategies for hospital-wide integration of the ICLN role. ICPs tested these materials in their respective hospitals. To explore their experiences with both the application of materials and the collaborative process, focus group interviews were conducted. Normalization Process Theory (NPT) was used as a framework to guide the analyses and the collaborative process itself.

**Results:**

Participants agreed upon the materials, but the extend to which their content became normalized in daily practice varied. In the interviews, ICPs mentioned that adoption and application of the materials depended on the implementation phase of the ICLN role. The collaborative process increased their confidence and intention to actively support ICLNs. It also helped them reflect on how to position the role within the organization, prompting them to consider various actions to embed the role structurally. Blueprint materials were considered helpful and provided practical strategies and hands-on activities and could be tailored to the local context.

**Conclusions:**

The collaborative process resulted in three practical, adaptable blueprint materials. The process helped ICPs reassess their own role in implementing the ICLN role, refine their training strategies, and strengthen their support for ICLNs at the ward level. Signs of normalization of the ICLN role varied across hospitals, influenced by both the stage of implementation and how ICPs interpreted and enacted their own role.

**Supplementary Information:**

The online version contains supplementary material available at 10.1186/s43058-026-00942-x.

Contributions to the literature

- Link nurses’ implementation of infection prevention requires training, tailored support and broad role integration, reflecting implementation science’s focus on context.
- We co-created materials to facilitate infection control practitioners (ICPs) in embedding this role in hospitals, contributing to the collection of resources that assist in normalization of evidence-informed interventions.
- While the developed materials themselves were important, it was the collaborative process that supported ICPs in clarifying their own role, contributing to gradual normalization of the link nurse role within their hospitals.
- These insights contribute to discussions on balancing the creation of user-friendly tools with the need to preserve implementation rigor.

## Background

The global burden of healthcare associated infections is high and antimicrobial resistance will continue to increase, unless we effectively engage health care professionals in the uptake of infection prevention measures [1, 2]. To improve the application of these measures, many infection prevention and control teams collaborate with infection control link nurses (ICLNs), who act as a link between colleagues in their own hospital department and the infection prevention and control team. ICLNs are typically volunteers or nominated by ward managers. Ideal candidates are assertive, responsible, enthusiastic about infection prevention, skilled in teaching and communication, and preferably full-time nurses with at least 1–2 years of patient care experience. Less experienced nurses with limited authority are generally considered unsuitable for the role [3]. The aim of ICLNs is to help improve the application of infection prevention and control measures through various strategies, such as motivating their peers to improve practice, translating policies into more practical work instructions, or sharing knowledge about infection prevention in clinical lessons [3, 4]. To fulfil this role, link nurses are trained by infection control practitioners (ICPs). In the Dutch context, ICPs are post-graduate professionals, usually with a background in nursing or trained as medical microbiology laboratory analysts. ICP teams distribute of responsibility among their members with one ICP often taking the lead supporting ICLNs.

The effectiveness of ICLNs can be improved in ways that are not unique to this model only, but are commonly applied in implementing innovations in practice [5]: a well-defined and clear role profile, commitment from the infection prevention and control team, support form the ward management, education on infection prevention topics in combination with training in soft skills and interaction with other ICLNs [3, 6–11]. At the ward level, action plans help to guide ICLNs’ activities, supporting a cyclic process of improvement through continuous reflection, adaption, and refinement of practices [3, 12]. To ensure the integration of the link nurse role within the hospital, two types of strategies can support implementation: the development of stakeholder interrelationships to drive cultural change and the use of evaluative and iterative strategies to manage system change [12, 13]. ICPs that build strong relationships can foster a shared responsibility for infection control. Combining bottom-up and top-down approaches helps ICPs to promote and integrate the link nurse role into all levels of the hospital organization, ensuring that link nurses receive the necessary support and backup to fulfil their role effectively [3, 12]. Additionally, active support from hospital management is crucial for implementing and sustaining the ICLN role. This support includes allocating necessary recourses and ensuring ongoing commitment through regular communication, training opportunities, and organizational backing. Continuous training helps ICLNs to take up their role better. An iterative approach, such as regular audits and feedback, enables ICPs to collaborate with ICLNs [14]. This collaboration strengthens two-way communication and helps refine responsibilities. It also supports the development of interventions that work best in their specific context. Finally, collaboration enables focus on addressing the most pressing infection prevention issues [3, 12].

Without clear guidance on how to support link nurses and integrate this role within hospitals, ICPs who train and assist ICLNs often develop their own ad hoc, unsystematic approaches locally, based on personal experiences and what is considered appropriate or most effective given the context. This results in significant variation in the content and structure of ICLNs support and the ICLN role. Consequently, ICPs may not include all essential elements or best practices, leading to role ambiguity for ICLNs, resulting in inconsistent and limited success despite investment of time and effort [6]. Typically, ICPs focus on educating link nurses and facilitating the transfer of knowledge [6]. This knowledge is essential for link nurses to adapt and integrate infection prevention measures within their ward’s specific context, and to identify infection prevention risks in order to address these or discuss solutions with an ICP.

However, successful implementation requires more than just education [6]. It is essential to discuss how infection prevention measures align with or disrupt existing workflows and to help ICLNs operationalize protocols [15]. This ensures a balance between safety and efficiency and effective integration in daily practice. For this, ICLNs need opportunities to engage in peer discussions about this operationalization, allowing them to exchange experiences and refine their approaches [15]. In addition to infection prevention knowledge, soft skills are crucial for link nurses to effectively communicate and drive infection prevention efforts within their wards. Strong interpersonal skills help them build relationships with peers, gain support, and engage hospital management, imperative for securing structural support and fostering a culture of shared responsibility [15, 16].

This heavy reliance on knowledge transfer – often described as the ‘train and pray’ approach in implementation science – underscores a common pitfall where practices depend heavily on training, assuming its effectiveness without ensuring its application and integration into practice [17]. Moreover, ICPs often overlook the broader implementation process and strategies needed to embed the link nurse role into everyday hospital practice [18]. As a result, training becomes an isolated objective rather than a tool for achieving the lagers goal of seamlessly integrating infection prevention measures into daily routines. Addressing this gap is crucial to enhance the real-world impact of ICLNs to fulfil their primary objective of strengthening infection prevention across hospitals.

To support ICLNs in successfully improving the application of infection prevention measures, and to move beyond mere knowledge transfer, ICPs need the tools to provide practical training and individualize support for link nurses. Additionally, helping ICPs to apply broader implementation strategies is essential to secure commitment from hospital and ward management, ensuring that the ICLN role is integrated and sustained within the hospital.

Recognizing these needs, we aimed to co-create blueprint materials for implementing the ICLN role and providing effective support for ICLNs. Co-creation is a collaborative, stakeholder driven process of creative problem solving were stakeholders and researchers participate as equal partners throughout all stages, from problem identification to implementation and evaluation [19]. In this study, co-creation was applied flexibly as an overarching guiding principle, emphasizing mutual respect, promoting equal participation, shared decision making and joint ownership, with the aim of enhancing the relevance of the blueprint materials and strengthening the impact of this collaborative process within the participating hospitals. In parallel, we sought to evaluate the real-world applicability of blueprint materials and examine how this collaborative process contributed to the support and normalization of the ICLN role across different hospital settings. Our research questions included: How do participants navigate the process of integrating information from the blueprint materials into their daily practice? And how did this process influence the normalization of the ICLN role?

## Methods

### Study design and theoretical framework

We used a qualitative research design and employed an action research approach grounded in principles of co-creation and user-centred design between October 2022 and May 2024 [19, 20].

To select and further develop materials already used in hospitals to support the implementation of the ICLN role, we collaboratively worked with stakeholders in eight 4-h sessions to developpractical, context specific solutions by jointly combining and supplementing useful elements from the existing materials into blueprint materials that could be tested between sessions [19]. This collaborative approach also enabled the implementation of changes within participants’ hospitals with feedback being used to iteratively refine the blueprint materials, taking into account the varying stages of implementation and specific contexts of each hospital. Based on the phase of the process and the input provided by stakeholders, the research team prepared the sessions employing various interactive formats, such as brainstorming, mind mapping, stakeholder mapping and sorting exercises, to encourage collaboration and structure idea generation [21]. Brainstorming was used to elicit perspectives and experiences related to the implementation of the ICLN role and supporting ICLN, as well as to generate ideas about the materials and their intended use. Mind mapping enabled participants to visually organise and connect emerging ideas, helping to identify connections between content, implementation challenges and potential solutions. Stakeholder mapping was employed to systematically identify relevant actors involved in or affected by the ICLN role, and to explore their roles and influences on its implementation. Sorting exercises were used to prioritize content and clarify preferences regarding format of the materials. Between session one and two context analyses were conducted to examine the roles and relationships involved in supporting ICLNs to ensure blueprint materials aligned with practical and organisational realities. A detailed overview of which methods were applied in each session, and how they were used is provided in Additional file 1. This approach ensured that the materials were tailored to panel members ‘ needs and aligned organizational dynamics [20].

The collaborative process was informed by the Normalization Process Theory (NPT) [22]. NPT is a middle-range sociological theory widely used in implementation science to understand how new interventions become embedded, or “normalized”, in routine practice. It focuses on the social processes through which individuals and groups make sense of an intervention (coherence), engage with it (cognitive participation), enact it in their daily work (collective action), and appraise its ongoing value (reflexive monitoring). NPT is therefore particularly well suited to studies seeking to understand and support the embedding of new practices within complex organizational settings such as hospitals. NPT was selected because this study did not merely aim to develop blueprint materials, but also to understand and support how the ICLN role becomes embedded in routine hospital practice. The ICLN role constitutes a complex intervention that requires changes in professional roles, interprofessional collaboration and organizational routines. NPT provides a theory-informed framework for examining how such changes are implemented an sustained through every day social processes. Its four generative mechanisms (coherence, cognitive participation, collective action, and reflexive monitoring) offered a structured way to link theory to our study objectives [23].

The collaborative sessions sought to build a shared understanding of the ICLN role (coherence), strengthen ICPs’ commitment and engagement (cognitive participation), and support concrete implementation efforts through co-created blueprint materials (collective action). Reflective discussions and focus group interviews enabled participants to assess and refine these efforts over time (reflexive monitoring).

NPT thus functioned both as a sensitizing framework to guide the collaborative process and as an analytic lens for interpreting how normalization unfolded across participating hospitals. Its focus on routinization in real-world settings makes it particularly appropriate for hospital contexts, where new roles must be integrated into established professional hierarchies, workflows, and governance structures. Applying NPT throughout the study allowed us to move beyond a descriptive account of material development and instead systematically examine how the collaborative process facilitated—or constrained—the embedding of the ICLN role in practice.

To ensure transparent and comprehensive reporting of out findings, we adhered to the Consolidated Criteria for Reporting Qualitative Research and the Standards for Quality Improvement Reporting Excellence 2.0 (Additional file I)[24, 25].

### Setting and participants

To form a panel, we identified and invited key stakeholders including ICPs (end-users), ICLNs, patient representatives, trainers, and researchers. Participation of these stakeholders was perceived important to the collaborative process as it allowed participants to review input for the blueprint materials from diverse perspectives, thereby aiming to achieve the broadest possible support for corresponding strategies. ICPs from Dutch acute care hospitals were recruited via the website of their national professional association, the Society for Hygiene and Infection Prevention in Healthcare (VHIG). In two-thirds of Dutch hospitals (n = 74), ICLNs are active [6]. We received responses from ICPs from 15 hospitals, of which ten provided a letter of commitment to participate: three academic medical centres, five specialized regional centres and two general hospitals. These ICPs were invited by the researcher (MD) to join the collaborative sessions and to recruit at least one link nurse from their respective hospitals. Additionally, we reached out to patient representatives through the client council of one hospital, as well as a policy advisor from the same institution. Participating hospitals compensated ICPs for their time and involvement in the project. Patient representatives and ICLNs who were not compensated by their hospitals received a €50 gift voucher and reimbursement for travel expenses for each collaborative.

session.

### Development of the blueprint materials

First, a panel consisting of stakeholders and researchers was formed to develop the materials. An initial session familiarized panel members with the project and explored their experiences with ICLNs and infection prevention. Panel members discussed both facilitating and hindering factors for successful ICLNs’ support, shared their expectations for the project, and identified their needs regarding the materials to be developed.

Then, the panel started collecting information about the context of each participating hospital; which elements were present to support ICLNs, what actions were planned, and what information, materials and tools (such as project plans, training outlines, Quick Scan formats, and meeting minutes) were being used. These elements were compared and discussed within subgroups organized by role to identify valuable components. During subsequent joint reflective discussion, panel members collectively decided which materials should be developed further and what content would be most impactful for implementing and supporting the ICLN role. This information was integrated by panel members to create materials. Subsequent sessions focused on refining and testing these materials. Panel members reflected on their implementation efforts, on the use of these materials in daily practice and commented on the content, navigation and layout of materials leading to iterative improvement of the materials through various versions.

Simultaneously, a context analyses and stakeholder mapping was conducted in each hospital to understand the roles and relationships involved in supporting ICLNs (Additional file I). Barriers to implement and sustain the link nurse role were defined and synthesized. These barriers, as well as additional barriers identified in previous work, were linked by the researcher (MD) to implementation strategies using the Consolidated Framework for Implementation Research—Expert Recommendations for Implementing Change Implementation Strategy Matching Tool and subsequently presented to the rest of the panel for feedback [12, 13, 26].

Finally, the impact of our approach was assessed on the adoption, implementation and sustainment of key elements to support ICLNs and implementation strategies to support the embedding of the link nurse role.

At the beginning and end of each collaborative session, reflective discussions were held both within the panel and within the research team. The panel reflections focused on the draft materials, their possible application within panel members’ own hospitals, on what they needed to further support implementation. During these moments, the exchange of experiences and best practices was actively encouraged. The reflections within the research team examined our facilitation approaches, assumptions and how our roles influenced group dynamics and decision making. This dual reflective practice enabled prioritizing the focus and format for subsequent sessions.

To prepare and facilitate active participation, panel members received agendas and draft materials before each session. Throughout the project, newsletters were sent to keep everyone informed. Between sessions, panel members received emails with interim assignments that included instructions for testing the materials and implementing its content, along with the offer of support form the researcher (MD).

### Data collection

During each collaborative session, detailed notes were taken consisting of summaries of discussions, observations, reflections from panel members, and their remarks. These notes were taken by the two researchers, who were not facilitating the session at that time (IJ, RM) or a panel member (SK). The observations primarily focused on group dynamics, indications of active engagement, such as participants’ contributions and, interactions in relation to their implementation activities. During the 5th and 7th session, panel members were asked to rate their confidence in supporting ICLNs and implementing the link nurse role on a walking scale from 0 (no confidence) to 10 (highly confident). They also reflected on if and how the materials had contributed to their confidence and skills set. After the final collaborative session, we conducted three focus group interviews with five, six and six participants respectively. The participants, including ICLNs, ICPs, and researchers, were divided in three groups that were not role-specific. Participants from the same hospital were distributed across groups to ensure that everyone could speak freely from their own perspective and experience. The interviews lasted 45 min each. Interviewers (MD, RM, IJ) were familiar with the interview guide and trained in interview techniques. The interview guide was informed by NPT [27]. We chose NPT as a lens to 1) capture and categorize the experiences of our panel members in implementing the ICLN role while co-creating and testing materials, and 2) to provide actionable recommendations for future adoption and implementation efforts. The topic list, that highlights the four primary NPT social mechanisms, is presented in Additional file III.

### Data analysis

Focus group interviews were recorded and transcribed verbatim by two researchers (MD, IJ). Three researchers (MD, IJ, RM) independently reviewed the transcripts multiple times to familiarize themselves with the data and highlight relevant quotes. We then employed the Rigorous and Accelerated Data Reduction (RADaR) technique, which consists of four sequential steps, to analyse the interviews [28]. In the first step, data formatting, one researcher (MD) created a data table that included al collectively (MD, IJ, RM) highlighted quotes. In the second step, data coding, the quotes were independently coded by two researchers (MD, IJ) using the core constructs and components of the NPT, along with any new codes as necessary. Any discrepancies in coding were resolved through discussion. In the third step, data reduction, one researcher (MD) organized the quotes by code and prepared a reduced data table. Data were further condensed into shorter tables; quotes that did not contribute to answering the research question or were repetitive were discussed and removed using ‘tracked changes’ (MD, RM, IJ) retaining quotes for each code. Finally, one researcher (MD) compared the final reduced table to notes from the collaborative sessions to actively seek confirmation or contradictory insights to triangulate findings and discussed these findings within the research team. We identified illustrative quotes to support our key findings and mapped these findings to NPT constructs as a final step of our analysis.

### Ethical considerations

The Medical Ethics Committee of Amsterdam UMC, location VUmc, determined that the study did not fall under the Dutch Medical Research Involving Human Subjects Act, as panel members were not subject to medical procedures or required to adhere to specific behavioural rules (reference 2023.0052) [29]. All panel members were informed on the scientific objectives of the study and provided their informed consent. Research data were stored and analysed in compliance with the General Data Protection Regulation.

## Results

In total, 30 stakeholders participated in the collaborative sessions. The panel comprised 19 ICPs, eight ICLNs, two patient representatives, and one policy officer, alongside a trainer and three researchers from the research team. At the start of the project, ICPs of four participating hospitals had just recently assigned ICLNs, two had experienced a downfall in their activities due to the COVID-19 pandemic and were working on the recovery of the link nurse role, and four hospitals had been collaborating with ILCNs for a longer period of time. Reasons for participation in the project included wanting the learn from peers, getting inspired, receiving information and tools to enhance their own activities, and contributing to shared knowledge for broader national application. ICPs average attendance across all sessions was 7.3 out of eight sessions, with a range from six to eight.

### Development of the blueprint materials and its use in practice

In total, the panel developed three blueprint materials which were derived from the needs and wishes expressed by the group: a role profile for ICLNs, an ICLN training program, and a document outlining implementation strategies.

First, the panel focused on defining the role profile by clarifying the purpose the role serves. Then, the panel members shifted their attention to training, recognizing it as a fundamental aspect of equipping link nurses with the necessary knowledge. During each session, panel members reviewed their efforts to implement the link nurse role in their hospital and assessed the use of the materials developed so far, incorporating insights from the context analysis—which was conducted between sessions one and two—in each hospital to determine which stakeholders to involve in the process. In the final collaborative session, the panel analysed these efforts in relation to previous research on implementation strategies for ICLNs [12, 13, 26].

#### Role profile

The panel compared existing ICLN role profiles from their hospitals, discussed the purpose of the role and the responsibilities it should encompass, and combined that into a unified profile. The final version of this material describes the organizational position, tasks, and responsibilities of ICLNs, along with a supporting paragraph outlining ways in which the profile can assist the link nurse in communication their role responsibilities or support the ICP in securing embedment of the role within the hospital.

From the collaborative sessions panel members found it important to develop a profile that allowed adaption of the role to the specific goals and context of the local ICLN initiative. By emphasizing that described tasks and responsibilities could be omitted or adjusted, its adaptability was highlighted in the materials’ introduction text.

Some panel members mentioned, during the focus group interviews, that they had used the role profile to build support for the role among infection prevention committees, departments, and management and for aligning expectations and responsibilities with individual link nurses. Other ICPs adapted the role profile to their own hospital setting in collaboration with ICLNs from their hospital, or opted to wait until the entire project was completed despite the project’s goal of testing and implementation.

… We have primarily focused on defining the role of the ICLNs based on the material regarding the role. We did this in collaboration with link nurses from our hospital, who provided input and feedback… [ICP, recovering the link nurse role, focus group interview].

… Not really implemented or used the material regarding the role profile yet. First we want to have a clear view of what implementing the ICLN role entails. We want to implement all materials at once and avoid constantly making small adjustments or modifications… [ICP, sustaining the ICLN role, session 5].

#### Training program

During the sessions, based on existing local materials for training ICLNs and under the guidance of the trainer (SI), the emphasis gradually shifted from transferring knowledge towards supporting ICLNs in assuming their role. A training guide was co-created to facilitate interactive ICLN training sessions, particularly emphasizing the importance of stating and communication objectives and expectations regarding the link nurses and the incorporation of training elements focused on how link nurses could fulfil their role and how to seek assistance in doing so. The final version provides an overview of potential training objectives covering various infection prevention topics, addressing different knowledge, skills, and attitude aspects, which can be tailored by selecting appropriate work methods. It also contains a supporting paragraph outlining ways to develop training that focusses on both content (infection prevention) and skills in how to assume and fulfil the role effectively. As an example, a complete training on how to improve hand hygiene for ICLNs was added, ready for use.

During the project, ICPs reported that they had not yet applied much of the content from the material. However, upon further inquiry, it became evident that they were indeed incorporating certain components into their education and training, sometimes unconsciously applying them. In contrast, others thoughtfully considered their application of this blueprint materials in practice and recognized potential pitfalls.

… I have learned here not to just deliver information while teaching. I learned this here and have improved somewhat, but we have not yet incorporated this into our whole ICLN education and training program … [ICP, recently assigned ICLNs, session 8].

…. We developed a comprehensive local training program and set objectives based on the role profile. However, when organizing the training with my colleague, we found ourselves brainstorming ideas to keep it interactive while I learned here that it’s essential to first consider the goals and what we want the ICLNs to take away from this training, and then determine which activities align with those objectives. In retrospect, I tried to incorporate this approach, but I found myself quickly wanting to move forward with fun ideas… [ICP, recovering the link nurse role, focus group interview].

#### Implementation process and strategies

In the final sessions, panel members reflected on their efforts to implement the link nurse role in their hospitals, assessing both their experiences and the use of the material developed so far. Each ICP was asked to complete an Excel form in which they described the strategies they used – whether aimed at implementing the role or supporting link nurses. They described how each strategy was operationalized, the intended goal of strategies, and whether that goal was achieved. These forms were collected and consolidated by MD, who also supplemented the data with strategies drawn from existing literature [12]. From the discussion of this information, a summary was compiled (MD) and used as core content for the third blueprint material. Additionally, the panel discussed the process that typically precedes and follows the appointment of ICLNs outlining the strategies used at each stage. Through these discussions, the panel defined three stages in the implementation, detailing how ICPs achieve ideal progress: initiation, formalization, and consolidation. While these stages suggest a developmental trajectory, the panel recognized that implementation in practice is often non-linear and context-dependent, with hospitals and departments progressing though the stages in different orders- or circling back to earlier ones as needed.

The initiation stage involves collaboration between the ICP and a few nurses to address a specific infection prevention issue at the department level. The collaboration is typically initiated by the ICP, often triggered by audit results, outbreaks, or rising antimicrobial resistance. Success in this phase leads to support from department management, which can drive the expansion of the ICLN role to other hospital departments. In the formalization stage, hospital stakeholders (such as clinical microbiologists, infectious disease specialists, or quality and safety mangers) are engaged to secure formal support for the ICLN role. Key activities include developing a role profile, a training plan, and action plans for ICLN activities. Bringing ICLNs together to share experiences helps solidify their role within the hospital. In the consolidation stage, the ICLN role becomes embedded in the hospital’s structure. Maintaining active involvement from stakeholders and fostering strong relationships across departments ensures continued support. Ongoing communication, reporting on the impact of ICLN activities and periodic evaluations help to embed the role, and adjust activities as needed. The ICP plays a central role throughout, initially supporting and motivating nurses and later taking on a more structured role as facilitator. They are responsible for guiding ICLNs, and using effective implementation strategies to ensure support for the role.

The panel concluded that a description of this process should be added as a third material to the blueprint, as it provides guidance for identifying local barriers and selecting stage specific implementation strategies to embed the link nurse role within the hospital. Therefore, the final material features a schematic representation of the process of embedding the ICLN role along with applicable implementation strategies. For future scientific research, we summarized these findings into a logic model (Additional file IV).

During focus group interviews and collaborative sessions, panel members expressed varying ideas about the role of the ICP in the implementation process. Discussions focused on lobbying for the ICLN role, influencing stakeholders and providing feedback to management on participation of individual ICLNs. Some ICPs described a shift in their perspective – from primarily focusing on education and training of ICLNs to recognizing their potential in creating a more receptive organizational environment. They became increasingly aware of their ability to take more proactive steps in generating hospital wide urgency and support for the ICLN role and advocating for its formal integration.

… I completely agree that you should do it (provide feedback), but it just doesn’t fit with our culture. Discussing it here really makes me think about whether I ultimately want to apply that strategy or not … [ICP, sustaining the ICLN role, focus group interview].

… I recall that in the second-to-last meeting, we had extensive discussions about communication both upwards and downwards within the organization. We, as ICPs, really need to make that step, and I recognize that as well… ensuring sustainability of the role… [ICP, recovering the link nurse role, session 8].

### Reflection of panel members on the value of the collaborative sessions

Overall, panel members found the concept of the collaborative sessions valuable and agreed on the importance of continuing these sessions. Some reflections on the sessions indicated a tendency to emphasize individual learning over the collective objective. Nevertheless, panel members recognized their collaborative efforts in developing a shared understanding of what implementing the ICLN role involves through their contributions to the blueprint materials. They emphasized that discussing best practices was vital in strengthening this collective understanding.

… I’ve heard many people say things that made me think, "Oh, that’s interesting! I’ve never considered that." … When it’s just the ICPs from our hospital, you tend to stay within a certain circle of discussion, but now we’re getting many different perspectives. For instance, how are other hospitals handling certain things that haven’t been implemented yet? Your horizon really broadens… [ICP, sustaining the ICLN role, focus group interview.

….the fact that there are ready-to-use products available is very successful—not just for us. I can definitely see what I can take from that and also show my colleagues, as they can benefit from it also … it has a lot of value for colleagues across the country… [ICP, sustaining the ICLN role, focus group interview.

In addition to the remarks on the value of the sessions, there was a discussion about organizing follow-up meetings or further developing meetings for all Dutch ICPs to join. Participants had already identified additional topics, such as monitoring and evaluating effects, for a fourth blueprint material. Several ICPs needed to consult their organizations before committing to continued participation. While many contributed ideas for how the sessions could continue, they did not immediately see themselves as the designated party for sustaining the sessions.

### Summary of key findings

Table 1 summarizes the results, of the collaborative sessions, showing how ICPs engaged in the project and how their efforts contributed to the normalization of the ICLN role within their hospital contexts, aligned with NPT constructs.

**Table 1:** Summary of key findings in relation to the NPT constructs

NPT construct	Theme in co-creation	Theme in normalization within own context
Coherence	Participants joined the project the project to learn from peers, to get inspired, to receiving information and tools to enhance their own practices, and to contribute to a national shared knowledge base Topics for blueprint development were derived directly from the participants needs and prioritized accordingly	ICPs showed varied understanding of their role. Some reported a shift in their perspective, from focusing on education and training of ICLNs to recognizing their potential in fostering a hospital wide culture receptive for the ICLN role. This included proactively creating a urgency and advocating for the role
Cognitive participation	Participants were deeply engaged throughout the co-creation, consistently attending sessions and actively contributing to discussions and material development	ICPs engaged in discussions about the materials with their local ICLNs, infection prevention peers and other stakeholders such as members of the Infection Control Committee to ensure alignment and application within their local context
Collective action	Participants collaborated to develop blueprint materials, which were then tested, discussed and refined through shared deliberation	The application of the blueprint materials varied depending on phase of ICLN role implementation and individual ICP perspectives on how to adapt and adopt them. This variability reflected differences in local contexts and perceptions of ICPs in their ideas about their own role in the implementation process
Reflexive monitoring	Panel members collectively evaluated their experiences, noting that discussing best practices strengthened their shared understanding of what implementing the ICLN role involves	ICP reflected differently on their contributions and impact, with some reassessing their role in fostering normalization. This led to refinements in strategy and further adaptation of the materials to better suit their local settings

## Discussion

Our study findings, framed through the Normalization Process Theory (NPT), demonstrate how participants’ initial motivations shaped the development of adaptable blueprint materials that addressed their practical needs. Despite the iterative approach and the emphasis on adapting strategies to improve and sustain implementation, a key finding was the extent to which the link nurse role appeared to be moving towards normalization in daily practice [30]. In line with findings from Bornbaum et al. and Santos and colleagues, normalization varied across participants’ hospitals and reflected the diversity in ICPs evolving understanding of their role as implementation champions, from primarily educating ICLNs to actively fostering a hospital wide culture supportive of the ICLN role [31, 32]. Some ICPs actions reflect both normative restructuring (adjusting workplace norms to align new practices with professional standards) and relational restructuring, which involves redefining roles and fostering collaboration[33]. We found that ICPs perception of their role and responsibilities closely tied to how actively they initiated these changes. Effective implementation of the ICLN role, and the creation of a sustainable system to support it at both organizational and individual levels ultimately depends on structural and behavioural changes [34]. ICPs are well-positioned to lead these efforts, particularly through systematic stakeholder identification and engagement [35]. Although the collaborative sessions were valuable, additional support and training tailored to the needs of individual IPCs are required. This ensures they are fully equipped with the essential competencies to lead the normalization process of the ICLN role and to recognize the importance of these skills for successful implementation [36, 37].

Also important in our findings was the recognition that, while stakeholder mapping (Additional file I) was an activity during the project, a more structured support to help ICPs map stakeholders in their own setting may have helped ICPs to fully recognize the importance of broader stakeholder engagement in implementation of the ICLN role while strengthening their restructuring skills. Using a stakeholder salience framework for instance, considering power, legitimacy, and urgency, could have increased ICPs awareness of the need to secure stronger commitments [35, 38]. Probably, a more comprehensive stakeholder analysis is needed to better equip ICPs to enhance their role as internal facilitators [39, 40]. However, the collaborative approach used in this project deliberately prioritized participant-driven discussions and adaptive learning, which shaped both the topics covered as well as the learning pace. Consequently, topics important for normalization, as well as knowledge gaps, are not always prioritized when participants collectively set the agenda [41]. This is exactly what we observed. Substantial attention was devoted to training ICLNs, a topic considered highly important by ICPs, leaving less time for a structured stakeholder analysis, which should be addressed in future initiatives.

Participants were highly engaged in the project, as demonstrated by their active contributions and consisted attendance during the sessions. Despite this enthusiasm and a shared interest in contributing some form of the supportive environment after the project ended, it did not lead to a sustained continuation of joint activities. This mirrors similar dynamics observed within hospital-based ICLN initiatives, where intervision sessions and opportunities for peer exchange often need to be facilitated by ICPs, even though ICLNs themselves express a clear desire to share best practices [15]. Sustaining these sessions was not a goal of this research project and should therefore not necessarily be considered a negative outcome. In retrospect, a key factor in this limited long-term engagement may have been the nature of theses sessions, which may have leaned more towards external facilitation than to genuine co-creation. As this was a research-driven project, there was an imbalance in roles, with facilitation led by the research team, rather than shared equally among all participants. According to Eldh et al*.*, external facilitation involves a structured, guided approach rather than one that emerges organically through participant collaboration [19, 42]. While this method provides clear direction, it may not cultivate the same sense of ownership and sustained commitment that authentic co-creation fosters.

While the collaborative process did not fully functioned as originally intended – fostering equal and sustained collaborative ownership – certain directive strategies did facilitate progress. For instance, forming alliances and establishing a structured cycle of sense-making, action, and reflection helped strengthen collaboration and sustained engagement during the project [43]. These approaches equipped ICPs with the skills necessary to support ICLNs and normalize the link nurse role within their hospitals. Additionally, continuous feedback from the researchers to internal facilitators (ICPs) and shared reflections within the group, ensured ongoing support and refinement a every stage of the project [44]. For scaling and implementing the ICLN role, an external facilitated and structured approach, incorporating these key elements, would likely be more effective than relying solely on an open-ended collaborative model. Such facilitation can guide the process, maintain momentum, and embed sustainable practices, ultimately enhancing the impact of the ICLN role across hospitals.

### Strengths and limitations

Our sample of participants from ten hospitals across the Netherlands contributed diverse experiences, skills, and perspectives on supporting link nurses and embedding the ICLN role in various hospitals contexts, including academic medical centres, top clinical and regional hospitals.

The application of Normalization Process Theory throughout the collaborative process and a as a framework for focus group interviews offered a structured lens to explicitly explore the implementation challenges of this complex intervention. To enhance the robustness of our findings, we triangulated focus group data with observations and reflections integrated into the notes from the collaborative sessions. While generalizability is not the goal of qualitative research, the range of contexts and participants represented in our sample supports the broader relevance and applicability of our insights to similar efforts to strengthen the ICLN role in hospitals.

A key limitation of our study is that, although supported by colleagues who were not involved in the project, the focus group interviews were conducted by the researchers. We acknowledge that this may have influenced participants responses. We aimed to minimize this risk by discussing the researchers ‘ dual role beforehand. During the project, an open and trusting atmosphere developed, which facilitated active participation during collaborative sessions and focus group interviews. Importantly, participants also voiced critical reflections on the process including aspects they felt missing, suggesting they did not refrain from expressing concerns or divergent views.

An additional limitation is the nature of participant involvement, which leaned more towards external facilitation than to co-creation. This approach generated valuable insight, yet also shaped the type and depth of data collected, potentially reflecting externally steered contributions rather than jointly developed understandings. Throughout the project, we attempted to mitigate power dynamics by jointly deciding on the topics to be discussed and prioritizing these through collective voting, giving participants greater influence over the agenda. We also incorporated moments of reflection to acknowledge and reduce our potential dominance in steering conversations. While these strategies supported a more balanced participation, more systematic and continuous reflection on power would have strengthened our approach [45].

While this and previous studies have identified key implementation mechanisms, a more structured approach is necessary to refine these insights and validate their impact [46]. Our logic model offers a systematic way to measure relevant implementation outcomes, ensuring a clearer understanding of success drivers. Additionally, building on this study a realist evaluation could provide deeper insights into how and why specific factors influence the normalization of the ILCN role across different hospitals, helping to uncover underlying facilitation processes [47, 48].

## Conclusions

ICPs participating in this project became increasingly aware of the complexities and requirements involved in supporting ICLNs and implementing the role in hospitals. By working together, adaptable materials were developed for implementation and integration of the ICLN role within hospital settings. Despite challenges such as transitioning to interactive training and advocating for the role, the collaborative process contributed to ICPs’ confidence and fostered an emerging awareness of what is needed for normalization. While ICPs applied the materials in practice, offering insights into their real-world applicability, it was the collaborative development process, rather than the content of the materials itself, that the participants found most valuable.

For the long term integration of the ICLN role, sustained support for ICPs in applying these materials seems essential. The collaborative process was crucial in helping ICPs clarify and align their role in the normalization of the ICLN role in within their hospital. This process involved fostering discussions, sharing experiences, refining skills related to the role’s integration, and helping ICPs making sense of their own role and influence within their organization. To ensure continued success, this cycle of sense making, participation, action and reflexive monitoring should be supported by an external facilitator who can help maintain momentum, guide implementation, and promote cross hospital learning.

## Supplementary Information

Additional file 1.

Additional file 2.

Additional file 3.

Additional file 4.

## Data Availability

The final coding table (in Dutch) is available from the corresponding author upon reasonable request. The blueprint materials (written in Dutch) are available upon request from the corresponding author.

## Abbreviations

ICLN: Infection control link nurse
ICP: Infection control practitioner
NPT: Normalization Process Theory
RADaR: Rigorous and Accelerated Data Reduction technique
VHIG: The Society for Hygiene and Infection Prevention in Healthcare
