# Supplementary material for: Development of blueprint materials that strengthen and embed the infection control link nurse role in hospitals – an action research study
Source: Implement Sci Commun. 2026 Apr 18;7:106. doi: 10.1186/s43058-026-00942-x (PMC13220495; doi:10.1186/s43058-026-00942-x)
Supplement: Supplementary file 1 — Additional file 1. [file 43058_2026_942_MOESM1_ESM.docx]

**ADDITIONAL FILE I** Project overview

| **Co-creation session, date** | **Session emphasis** | **Session Formats** | **Short description of how the techniques were applied** | **Interim assignment** |
| --- | --- | --- | --- | --- |
| 1, September 2022 | Acquaintance and expectations  Introduction to project  Perspectives on important elements of support for the ICLN role | Brainstorming  Sorting exercise | Panel members generated ideas in small groups using brainstorming.  These ideas were then discussed within the whole group and prioritized and sequenced according to their importance and logical order. | Discuss elements of support for the ICLN role and prioritization of this support in own hospital |
| Activity in between: Master students from the Medisign program at Delft University of Technology conducted context and stakeholder mapping conducted at each hospital. Using the SEIPS 2.0 framework, a socio-technical systems approach, they created work system diagrams to visualize each hospital’s system. The students collaborated with panel members through site visits and stakeholder interviews. Their final output, presented to the panel, provided insights into the roles and relationships involved in supporting ICLNs. | | | | |
| 2, December 2022 | Experiences with context analysis and preparing for stakeholder analysis in own hospital  Preparing a generic role profile including support of ICLN | Group discussion  Mind mapping  Sorting exercise | Panel members reflected on the context and stakeholder mapping and shared their thoughts and experiences.  Panel members mapped elements relevant to support ICLN assuming their role    Panel members reordered elements from existing role profiles according to their relevance or appeal and discussed why these elements stood out. | Enrich or refine the stakeholder map of own hospital with input from this discussion  Discuss role profile and need for support of ICLN in own hospital |
| 3, March 2023 | Content of generic role profile ICLN  Perspectives on content of ICLN training | Group discussion  Brainstorming  Sorting exercise | Panel members reflected on the input they had gathered within their own hospital regarding the interim assignment.  Panel members discussed the optimal way to design education and training for ICLN to ensure alignment with the generic role profile  Panel members reordered elements from existing role profiles according to their relevance or appeal and discussed why these elements stood out. | Discuss ideal ICLN training in own hospital |
| 4, June 2023 | Discuss concept training  Perspectives on contextual factors that influence ICLN | Brainstorming  Brainstorming  Mind mapping | Panel members reordered elements from existing training programs according to their relevance or appeal and discussed why these elements stood out.  Panel members discussed the knowledge and skills required to facilitate such training and whether these align with the profile of an infection control practitioner  Panel members discussed factors that influence embedding and uptake of the ICNL role in hospitals  Panel members mapped factors that influence embedding and uptake of the ICNL role in hospitals to the SEIPS Work System Components, and described under each category, facilitating and inhibiting factors. | Discuss factors that influence embedding and uptake of the role in own hospital |
| 5, September 2023 | Experiences with interactive training elements  Discuss a training program for hand hygiene | Brainstorming  Mind mapping  Walking scale | Panel members reflected on the input they had gathered within their own hospital regarding the interim assignment.  Panel members reflected on the draft blueprint prototype of the role profile  Participants worked on an example training regarding hand hygiene, and mapped the required knowledge, skills, and attitude components based on the discussions in previous sessions.  Panel members rated their confidence in supporting ICLNs and implementing the link nurse role | Test elements in own hospital: what does (not) work?  Collaborate with panel members on training program for hand hygiene |
| 6, December 2023 | First impression of generic plan for ICLN training  Discuss strategies to implement the ICLN role: plans, opportunities and limitations | Brainstorming  Mind mapping | Panel members reflected on the input they had gathered within their own hospital regarding the interim assignment.  Panel members reflected on the draft blueprint prototype of the role profile and training program  Panel members discussed the elements that are needed to optimally support ILCN in hospitals other than training, more on an organizational level  Panel member discussed the barriers identified in session four in implementing and sustaining the link nurse role, and supporting ICL  Panel members mapped the actions they had take, the strategies they used, and the additional step they could take to overcome these barriers | Test training materials |
| 7, March 2024 | Agreement on generic training plan for ICLN  First overview of strategies and other elements that stakeholders currently apply | Brainstorming    Sorting exercise  Walking scale | Panel members reflected on the input they had gathered within their own hospital regarding the interim assignment.  Panel members reflected on the draft blueprint prototype of the role profile and training program  Panel members reordered elements from existing action plans, including actions taken in implementing and sustaining the link nurse role according to their relevance or appeal and discussed why these elements stood out.  Panel members rated their confidence in supporting ICLNs and implementing the link nurse role | Test and discuss materials in own hospital |
| 8, May 2024 | Discussion on how ICLN role is generally implemented | Brainstorming | Panel members reflected on the input they had gathered within their own hospital regarding the interim assignment.  Panel members reflected on the draft blueprint prototype outlining implementation strategies. |  |
