# Supplementary material for: Development of blueprint materials that strengthen and embed the infection control link nurse role in hospitals – an action research study
Source: Implement Sci Commun. 2026 Apr 18;7:106. doi: 10.1186/s43058-026-00942-x (PMC13220495; doi:10.1186/s43058-026-00942-x)
Supplement: Supplementary file 2 — Additional file 2. [file 43058_2026_942_MOESM2_ESM.docx]

**ADDITIONAL FILE I** Co-design project overview

| **Co-creation session, date** | **Session emphasis** | **Interim assignment** |
| --- | --- | --- |
| 1, September 2022 | Acquaintance and expectations  Introduction to project  Perspectives on important elements of support for the ICLN role | Discuss elements of support for the ICLN role and priority in own hospital |
| Activity in between: context and stakeholder analysis by students per hospital | | |
| 2, December 2022 | Experiences with context analysis and preparing for stakeholder analysis in own hospital  Preparing a generic role profile including support of ICLN | Discuss role profile and need for support of ICLN in own hospital |
| 3, March 2023 | Content of generic role profile ICLN  Perspectives on content of ICLN training | Discuss ideal ICLN training in own hospital  Discuss criteria for materials |
| 4, June 2023 | Discuss concept training  Perspectives on contextual factors that influence ICLN | Discuss factors that influence embedding and uptake of the role in own hospital |
| 5, September 2023 | Experiences with interactive training elements  Discuss a training program for hand hygiene | Test elements in own hospital: what does (not) work?  Collaborate with panel members on training program for hand hygiene |
| 6, December 2023 | First impression of generic plan for ICLN training  Discuss strategies to implement the ICLN role: plans, opportunities and limitations | Test training materials |
| 7, March 2024 | Agreement on generic training plan for ICLN  First overview of strategies and other elements that stakeholders currently apply | Test and discuss materials in own hospital |
| 8, May 2024 | Discussion on how ICLN role is generally implemented |  |
