# Supplementary material for: Development of blueprint materials that strengthen and embed the infection control link nurse role in hospitals – an action research study
Source: Implement Sci Commun. 2026 Apr 18;7:106. doi: 10.1186/s43058-026-00942-x (PMC13220495; doi:10.1186/s43058-026-00942-x)
Supplement: Supplementary file 3 — Additional file 3. [file 43058_2026_942_MOESM3_ESM.docx]

**ADDITIONAL FILE II** Topiclist

| **NPT Core Construct** | **Questions per topic** |
| --- | --- |
| **Coherence** is the sense-making work that people do individually and collectively when they are faced with the problem of operationalizing innovations and interventions**.** | **Coherence (Sense Making Work)**    What were your experiences with the link nurse role before participating in the project?  Can you describe the aim of that role and the key elements that support it?  In your opinion, what is the role of the infection prevention expert in implementing the link nurse role? |
| **Cognitive Participation** is the relational work that people do to build and sustain a community of practice around innovations or complex interventions. | **Cognitive Participation (Engagement Work)**   During this project, has anything changed in the interactions or communication - within your team? - with the link nurses within your hospital? - with other stakeholders in your hospital? How do you assess the impact of these changes? Were there any team members or stakeholders who played a significant role in the implementation process? How has the project shaped your perspective on your role and responsibilities in implementing the link nurse role? If so, have these responsibilities been allocated differently as a result? |
|  |  |
|  |  |
|  |  |
| **Collective Action**  is the operational work that people do to enact a set of practices, whether these represent an innovation or a complex intervention. | **Collective Action (Enacting Work)**  What were your experiences with the implementation of the link nurse role in your hospital during the project? What steps were taken and which elements did you use? What adjustments were made to fit the implementation phase or the local goals of your hospital? Why? Were any adjustments made to the goal of the role or the activities to support link nurses? Why? Which elements have you not used yet? Why? Have these experiences changed your opinion about the goal of the link nurse role and the way (referring to elements) to achieve that goal? Have these experiences impacted the way you execute activities to support link nurses within your hospital? |
|  |  |
|  |  |
|  |  |
| **Reflexive Monitoring** is the appraisal work that people do to assess and understand the ways that the innovation or complex intervention affect them and others around them. | **Reflexive Monitoring (Appraisal Work)**    To what extent do you now use the knowledge from the sessions in your daily work? Can you give examples of how you have turned knowledge from the sessions into action? What challenges have you solved regarding the implementation of the link nurse role? What challenges are you still facing? What do you need to address these challenges? |
|  |  |
|  |  |
|  |  |
| **The contributions of co-creation…** | What were the most valuable aspects of the codesign sessions for you? |
|  | **…to dissemination** How effective were the sessions in sharing knowledge about the implementation of the link nurse role? |
|  | What were the most valuable aspects of the sessions for you? Were there any aspects of the sessions that could be improved? |
|  | **…to implementation** How did the knowledge shared during sessions influence the implementation process? Can you describe any changes in practice or decision-making that resulted from these sessions? |
| **Closing** | Is there anything else you would like to add about your experience with the ICLN role or the sessions? Do you have any suggestions for improving the implementation process or the sessions in the future? |
