# Supplementary material for: Development of blueprint materials that strengthen and embed the infection control link nurse role in hospitals – an action research study
Source: Implement Sci Commun. 2026 Apr 18;7:106. doi: 10.1186/s43058-026-00942-x (PMC13220495; doi:10.1186/s43058-026-00942-x)
Supplement: Supplementary file 4 — Additional file 4. [file 43058_2026_942_MOESM4_ESM.docx]

**ADDITIONAL FILE III** Logic model

**Caption**. The importance of different activities shifts throughout the stages of implementation. The lighter the shading, the earlier these elements and strategies are applied,

while the darker (grayer) the shading, the later they come into play in the process.
